# A Double Triage and Telemedicine Protocol to Optimize Infection Control in an Emergency Department in Taiwan During the COVID-19 Pandemic: Retrospective Feasibility Study

**DOI:** 10.2196/20586

**Published:** 2020-06-23

**Authors:** Chien-Hao Lin, Wen-Pin Tseng, Jhong-Lin Wu, Joyce Tay, Ming-Tai Cheng, Hooi-Nee Ong, Hao-Yang Lin, Yi-Ying Chen, Chih-Hsien Wu, Jiun-Wei Chen, Shey-Ying Chen, Chang-Chuan Chan, Chien-Hua Huang, Shyr-Chyr Chen

**Affiliations:** 1 Department of Emergency Medicine National Taiwan University Hospital National Taiwan University College of Medicine Taipei Taiwan; 2 Institute of Occupational Medicine and Industrial Hygiene College of Public Health National Taiwan University Taipei Taiwan

**Keywords:** COVID-19, triage, emergency department, health care workers, infection control, telemedicine

## Abstract

**Background:**

Frontline health care workers, including physicians, are at high risk of contracting coronavirus disease (COVID-19) owing to their exposure to patients suspected of having COVID-19.

**Objective:**

The aim of this study was to evaluate the benefits and feasibility of a double triage and telemedicine protocol in improving infection control in the emergency department (ED).

**Methods:**

In this retrospective study, we recruited patients aged ≥20 years referred to the ED of the National Taiwan University Hospital between March 1 and April 30, 2020. A double triage and telemedicine protocol was developed to triage suggested COVID-19 cases and minimize health workers’ exposure to this disease. We categorized patients attending video interviews into a telemedicine group and patients experiencing face-to-face interviews into a conventional group. A questionnaire was used to assess how patients perceived the quality of the interviews and their communication with physicians as well as perceptions of stress, discrimination, and privacy. Each question was evaluated using a 5-point Likert scale. Physicians’ total exposure time and total evaluation time were treated as primary outcomes, and the mean scores of the questions were treated as secondary outcomes.

**Results:**

The final sample included 198 patients, including 93 cases (47.0%) in the telemedicine group and 105 cases (53.0%) in the conventional group. The total exposure time in the telemedicine group was significantly shorter than that in the conventional group (4.7 minutes vs 8.9 minutes, *P*<.001), whereas the total evaluation time in the telemedicine group was significantly longer than that in the conventional group (12.2 minutes vs 8.9 minutes, *P*<.001). After controlling for potential confounders, the total exposure time in the telemedicine group was 4.6 minutes shorter than that in the conventional group (95% CI −5.7 to −3.5, *P*<.001), whereas the total evaluation time in the telemedicine group was 2.8 minutes longer than that in the conventional group (95% CI −1.6 to −4.0, *P*<.001). The mean scores of the patient questionnaire were high in both groups (4.5/5 to 4.7/5 points).

**Conclusions:**

The implementation of the double triage and telemedicine protocol in the ED during the COVID-19 pandemic has high potential to improve infection control.

## Introduction

### Background

Since the beginning of the 20th century, various infectious diseases have repeatedly threatened both population health and health care systems worldwide. The rapid growth of international transportation has paved the way for the transmission of infectious diseases across regions, including severe acute respiratory syndrome (SARS), H1N1 influenza, Middle Eastern respiratory syndrome (MERS), and Ebola virus [1-4]. In 2020, coronavirus disease (COVID-19) was first reported in Wuhan, China; this virus spread globally through large-scale transmission and continues to pose great challenges to medical, public health, and socioeconomic systems worldwide [5]. Severe acute respiratory syndrome coronavirus 2 (SARS-CoV-2), identified as the causative pathogen of COVID-19, is highly contagious [6]. It is primarily transmitted through droplets and close contact, even in the early course of the disease, as well as from asymptomatic patients [6]. The response strategies for controlling COVID-19 outbreaks include early diagnosis, patient isolation, symptomatic monitoring, and quarantine [7].

During the response to the COVID-19 pandemic, a considerable number of frontline health care workers have been infected with the new coronavirus. In China, the first COVID-19 case among health care workers was reported on January 20, 2020; as of February 11, 2020, health care workers represented 1716/44,672 (3.8%) of all patients with laboratory-confirmed COVID-19 and 247/1668 (14.8%) of critical cases, with 5 deaths [8]. In Italy, health care workers accounted for 2026/22,512 (9.0%) of confirmed cases as of March 15, 2020 [9]. According to a recent report from the US Centers for Disease Control and Prevention (CDC), this figure in the United States was 9282/315,531 (2.9%); 2.1%-4.9% of health care workers required intensive care unit admission, and 27 deaths were reported [10]. By April 8, 2020, COVID-19 had affected 22,073 health care workers in 52 countries [11]. The transmission of SARS-CoV-2 among frontline health care workers may result from their long, direct exposure to many infected patients and from a lack of personal protective equipment (PPE) [12]. Therefore, further infection control measures should be undertaken to minimize the direct exposure of health care workers to patients suspected of having COVID-19 [7].

### Importance

Emergency departments (EDs) are at the frontline of the health care response to the COVID-19 pandemic and are responsible for rapid and safe triage of patients and isolation of suspected patients with COVID-19 from patients with noninfectious diseases. Therefore, establishing a screening protocol for patients with suspected COVID-19 and isolating them in a separate, well-ventilated space for clinical interviews can facilitate infection control [7]. Moreover, due to the shortages of PPE worldwide, its use should be optimized and prioritized based on critical requirements at health care facilities. Additional strategies to minimize exposure risk, such as implementing alternatives to face-to-face triage and hospital visits, can not only prevent health care workers from being exposed to COVID-19 but can also reduce PPE use. The combination of a specialized triage approach and telemedicine may be an alternative to reach this goal.

Telemedicine can be defined broadly as the use of telecommunications technology to provide medical information and services [13]. With the evolution in technology and online services, telemedicine may prove a compelling alternative to conventional acute, chronic, and preventive care [14-17]. Telemedicine, particularly video consultations, has been promoted to reduce the risk of disease transmission [18-23]. Certain reports have recently described potential benefits and applications of telemedicine during the COVID-19 pandemic [24-27]. However, the effectiveness of telemedicine in improving infection control has not been well studied, as most evidence has been obtained from clinical cases, and some studies merely propose theoretical assumptions.

### Goal of This Study

As of May 2, 2020, Taiwan had 432 confirmed COVID-19 cases, and a total of 63,713 patients were tested. Nearly 97% of the Taiwanese population have access to the health care system and are covered by the National Health Insurance; hence, they can readily access health care [28]. EDs were the first units that responded to the COVID-19 pandemic in Taiwan, and most confirmed patients underwent screening and evaluation in EDs [29]. To provide essential care with optimized infection control in the ED, a double triage and telemedicine protocol was developed at our hospital. This study was conducted to evaluate the benefits and feasibility of the novel protocol for reducing the risk of COVID-19 infection among health care workers who manage COVID-19 patients.

## Methods

### Study Setting and Design

The National Taiwan University Hospital (NTUH) is a 2700-bed teaching hospital that provides both primary and tertiary care. It was designated as a COVID-19 response hospital in Taiwan. From March to April 2020, the number of average daily visits was 211, and the number of average daily visits to the NTUH ED by patients posing a risk of COVID-19 transmission was 12.

We conducted a retrospective study using prospectively collected data of patients who visited the ED between March 1 and April 30, 2020. The study was approved by the Institutional Review Board of the NTUH (REC No. 202003043RINA), and the requirement to obtain informed consent was waived.

### The Double Triage and Telemedicine Protocol

**Figure 1:**
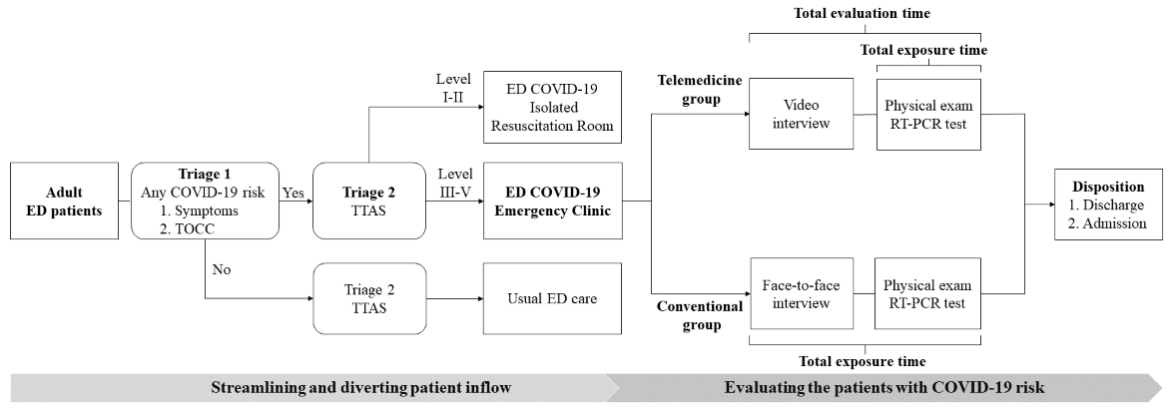
Diagram of the double triage and telemedicine protocol. COVID-19: coronavirus disease; ED: emergency department; TOCC: travel, occupation, contact, and cluster history; TTAS: Taiwan Triage and Acuity Scale.

In the course of the health care response to the COVID-19 pandemic, we developed a double triage and telemedicine protocol for the ED to manage patients suspected of having COVID-19. The protocol comprised two major components: 1) streamlining and diverting patient inflow using a double triage method and 2) evaluating suspected COVID-19 patients using telemedicine (Figure 1).

Regarding the double triage method, the first triage (Triage 1) was set up outside the entrance of the ED to screen patients based on their likelihood of posing a risk of COVID-19 transmission. The double triage helped examine the patients’ history of travel, occupation, contact, and cluster (TOCC) based on the guidelines issued by the Taiwan Centers for Disease Control and available epidemiological data [29]. The Triage 1 nurse used the Taiwan Triage and Acuity Scale (TTAS) to carry out a second triage (Triage 2) for patients who posed a risk of COVID-19 transmission to health care workers [30]. TTAS is a computerized triage system with a 5-level scale that classifies patients in descending order of acuity from Triage Level I (resuscitation) and Triage Level II (emergent) to Triage Level V (nonurgent). The TTAS assesses triage level according to existing chief complaints, vital signs (eg, degree of respiratory distress, hemodynamic stability, conscious level, change in body temperature, and pain severity), and mechanism of injury (for patients with trauma) to determine the triage level of a patient. The patients who triaged as TTAS level I usually had life-threatening diseases and needed immediate resuscitation. Those patients who triaged as TTAS level II usually had potential life-threatening diseases or organ dysfunction and needed management quickly. The recommended reassessment time was less than 10 minutes. For patients triaged as TTAS levels III to V, the recommended reassessment times were 30, 60, and 120 minutes, respectively. If a patient was triaged as TTAS Level I or II, they would be directed to an isolated COVID-19 resuscitation room for further management. Patients classified as TTAS levels III to V were referred to the COVID-19 emergency clinic, which is located in a structure that is separated from the main ED building. The COVID-19 resuscitation rooms and COVID-19 emergency clinics are equipped with new ED infrastructure that was constructed in response to emerging infectious diseases [31]. Patients with no risk of COVID-19 would be diverted by the Triage 1 nurse into an internal triage facility inside the ED and receive routine emergency care.

The health care workers in the COVID-19 emergency clinic were instructed to don appropriate PPE, including an N95 face mask, a waterproof gown, a non-disposable face shield, a hair cap, shoe sleeves, and two layers of gloves, when working in the clinic. A nurse was assigned to verify the appropriateness of the PPE and record the times of donning PPE, doffing PPE, and entering and exiting the clinic. In the COVID-19 emergency clinic, physicians performed face-to-face interviews with patients before performing physical examinations and collecting specimens for COVID-19 reverse transcription–polymerase chain reaction (RT-PCR) tests. In our study, patients evaluated using face-to-face interviews were assigned to the conventional group.

To improve infection control in the ED, we established a COVID-19 telemedicine team of nine attending physicians and four senior residents and provided them with training in the telemedicine interview system (TIS) on March 27, 2020. On April 1, 2020, the TIS was introduced in the COVID-19 emergency clinic (Multimedia Appendix 1) using U Meeting (CyberLink Corp) as the communication software. The TIS protects the privacy and security of patient data through end-to-end encryption and data transmission through an intranet governed by the firewall of the hospital’s information system. Furthermore, the TIS is in compliance with the government regulations for telemedicine. If a patient who was diverted to the COVID-19 emergency clinic agreed to attend a video interview, the patient’s attending physician on the COVID-19 telemedicine team would first use the TIS to conduct that interview. The physician would then share the patient’s chest radiography findings and disease information on a screen located at the patient’s side while explaining them to the patient. After the TIS interview, the physician would don appropriate PPE before entering the clinic to complete all the other necessary evaluations and obtain specimens for the COVID-19 RT-PCR test. We designated patients who underwent TIS evaluation as the telemedicine group. In both the telemedicine group and the conventional group, physicians would then decide the disposition of patients (ie, either discharging them or admitting them to quarantine wards).

The total exposure time was defined as the time that a physician remained in contact with a patient during their face-to-face interview. The total evaluation time was defined as the time the physician took to interview and assess the patient and collect specimens for laboratory testing. Both these time intervals were prospectively recorded.

Furthermore, we conducted a survey using a 10-item questionnaire in both groups upon completion of the clinical evaluation. The questionnaire was modified from a questionnaire for evaluating patient satisfaction with telemedicine [32]. It was also simplified to assess the immediate impression of the study participants because the survey was conducted in an isolated room and the response time was limited. The questionnaire helped us obtain information on the global rating of the interview, the quality of the interview, the mutual understanding between the patient and the physician, and perceptions of stress, discrimination, and safety. To assess the feasibility of telemedicine, we asked 3 additional questions to survey the participants’ acceptance, perception of safety, and satisfaction with the evaluation protocol. The level of agreement was assessed using the 5-point Likert scale, ranging from 1 point (extremely agree) to 5 points (extremely disagree). The questionnaire was validated by 4 emergency physicians and the director of the Center of Quality Management at the study center. Data on the patients’ admission, discharge, and clinical and personal backgrounds were obtained from the hospital’s health information system.

### Recruitment

We included adult patients aged ≥20 years who were diverted to our COVID-19 emergency clinic during the study period. Patients who refused TIS in the telemedicine group were excluded from this study. Furthermore, to control the confounding effect of evaluation by different physicians, patients not evaluated by the COVID-19 telemedicine team were excluded from the analysis dataset.

### Outcomes

The primary outcomes of interest included physicians’ total exposure time and total evaluation time. The secondary outcomes were the mean scores of the questionnaire survey used to evaluate the double triage and telemedicine protocol.

### Statistical Analysis

Descriptive statistics are presented as mean (SD) for continuous variables, and the intergroup differences in means were analyzed using the independent sample *t* test. The chi-square test was used to assess the associations between categorical variables, namely sex, marital status, education, TOCC history, chronic health conditions, and primary care physician level. To compare the total exposure time and total evaluation time between the two groups, we calculated their intergroup mean differences and 95% CIs. Multiple linear regression models were constructed to adjust for potential confounders and determine the effect of the telemedicine group on each of the outcomes. The identified covariates included age, sex, education, TTAS level, and primary care physician level. The goodness of fit for the multiple linear regression models was examined by computing the *R*^2^ statistic. Regression diagnostics were used to identify problems in the models or data. The answers to each questionnaire item were analyzed using the Mann-Whitney U test and are presented as mean scores. A two-tailed *P* value ≤.05 was considered to be statistically significant. All statistical analyses were conducted using SAS version 9.4 (SAS Institute).

## Results

### Characteristics of the Study Participants

**Figure 2:**
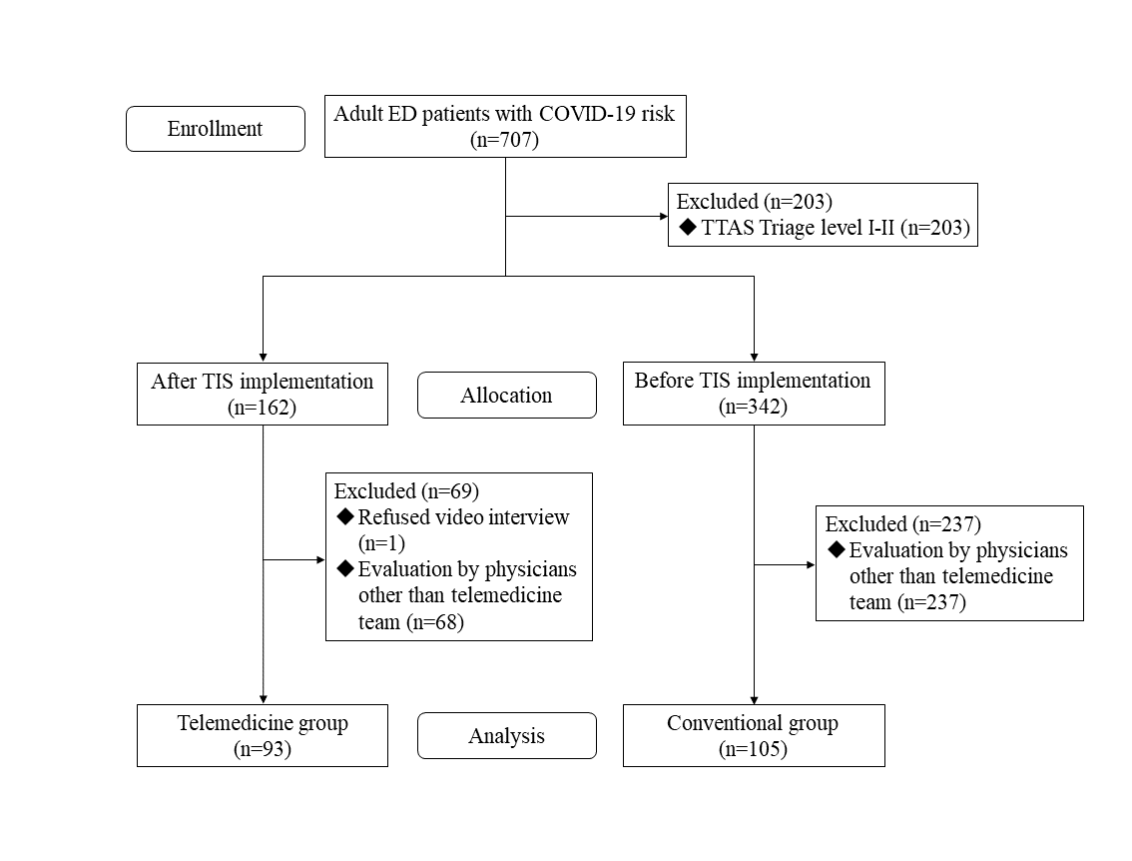
Flowchart of patient disposition. COVID-19: coronavirus disease; ED: emergency department; TTAS: Taiwan Triage and Acuity Scale.

We initially enrolled 707 adult ED patients in the study, of whom 203 (28.7%) were excluded from the study because of high TTAS levels (I and II). Before implementing the TIS, 342 patients were interviewed, of whom 237 (69.3%) were not interviewed by the telemedicine team. After the introduction of the TIS, 162 patients were interviewed, including 1 patient who refused the TIS and 68 others who were not interviewed by the telemedicine team. We also excluded all patients who were not interviewed by the telemedicine team and those who refused the TIS. Finally, 93 patients were included in the telemedicine group for analysis, compared with 105 patients in the conventional group (Figure 2).

The clinical characteristics of the patients are presented in Table 1. Compared to the conventional group, the telemedicine group had significantly higher rates of patients with an education level of senior high school or lower (31/93, 33.3%, vs 16/105, 15.2%; *P*=.003), TTAS triage level III (76/93, 81.7%, vs 58/105, 55.2%; *P*<.001), and cardiovascular diseases (8/93, 8.6%, vs 1/105, 1.0%; *P*=.014). The telemedicine group had a lower rate of patients with a travel history than the conventional group (26/93, 28.0%, vs 56/105, 53.3%; *P*<.001). The rate of resident doctors conducting interviews with patients in the telemedicine group was higher than that in the conventional group (39/93, 41.9%, vs 17/105, 16.2%; *P*<.001).

The mean age showed no significant difference between the telemedicine group and the conventional group (mean 39.8 years, SD 17.1, vs mean 38.4 years, SD 25.1; *P*=.65). The two groups did not show any difference in terms of the rate of male patients (41/93, 44.1%, in the telemedicine group vs 44/105, 41.9%, in the conventional group; *P*=.76) or that of married patients (22/93, 23.7%, in the telemedicine group vs 21/105, 20%, in the conventional group; *P*=.53). Regarding comorbidities, the rates of patients with diabetes (8/93, 8.6%, vs 2/105, 1.9%; *P*=.048), chronic renal disease (4/93, 4.3%, vs. 0/105, 0.0%; *P*=.047), and cardiovascular diseases (8/93, 8.6%, vs 1/105, 1.0%; *P*=.014) were significantly higher in the telemedicine group than in the conventional group. Meanwhile, no significant discrepancies were observed in the other rates.

**Table 1:** Baseline demographic and clinical characteristics of patients in the telemedicine and conventional groups (N=198).

Characteristic		Telemedicine group (n=93)	Conventional group (n=105)	*P* value
Age (years), mean (SD)		39.8 (17.1)	38.4 (25.1)	.65
Male gender, n (%)		41 (44.1)	44 (41.9)	.76
**Marital status, n (%)**				**.53**
	Married	22 (23.7)	21 (20.0)	
	Single, divorced, or widowed	71 (76.3)	84 (80.0)	
**Education, n (%)**				**.003**
	Senior high school or lower	31 (33.3)	16 (15.2)	
	University or higher	62 (66.7)	89 (84.8)	
**Epidemiologically significant TOCC^a^ history, n (%)**				
	Travel	26 (28.0)	56 (53.3)	<.001
	Occupation	36 (38.7)	41 (39.1)	.96
	Contact	19 (20.4)	25 (23.8)	.57
	Cluster	1 (1.1)	2 (1.9)	>.999
**ED^b^ triage level, n (%)**				**<.001**
	Triage level III	76 (81.7)	58 (55.2)	
	Triage level IV or V	17 (18.3)	47 (44.8)	
**Comorbidities, n (%)**				
	Diabetes	8 (8.6)	2 (1.9)	.05
	Malignancy	4 (4.3)	1 (1.0)	.19
	Chronic renal disease	4 (4.3)	0 (0.0)	.05
	Chronic liver disease	2 (2.2)	1 (1.0)	.60
	Cardiovascular diseases	8 (8.6)	1 (1.0)	.01
	COPD^c^ or asthma	3 (3.2)	5 (4.8)	.73
	Cerebrovascular accident	1 (1.1)	0 (0.0)	.47
	Hypertension	11 (11.8)	6 (5.7)	.13
**Primary care physician level, n (%)**				**<.001**
	Attending physician	54 (58.1)	88 (83.8)	
	Resident	39 (41.9)	17 (16.2)	
**ED disposition, n (%)**				**.06**
	Admission	20 (21.5)	35 (33.3)	
	Discharge	73 (78.5)	70 (66.7)	
72-hour ED revisit,^d^ n (%)		2 (2.7)	0 (0.0)	.50

^a^TOCC: travel/occupation/contact/cluster.

^b^ED: emergency department.

^c^COPD: chronic obstructive pulmonary disease.

^d^Hospitalized patients are excluded.

### Comparison of the Total Exposure Time, Total Evaluation Time, and 72-Hour Revisit Rate Between the Telemedicine Group and the Conventional Group

**Figure 3:**
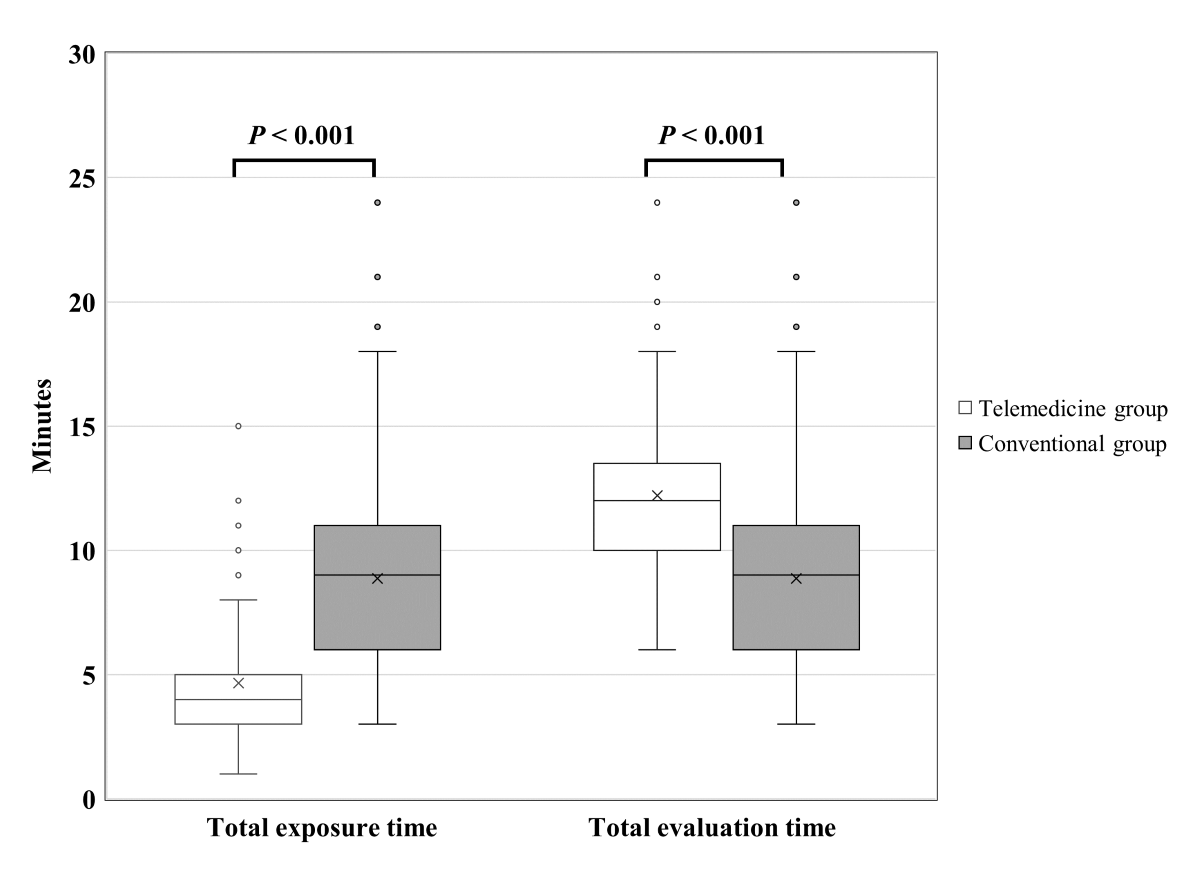
Box plot of the differences in the total exposure time and the total evaluation time between the telemedicine and conventional groups.

The total exposure time and total evaluation time were compared to estimate the benefits of the implementation of the double triage and telemedicine protocol (Figure 3). The total exposure time in the telemedicine group was significantly shorter than that in the conventional group (4.7 minutes, SD 2.4, vs. 8.9 minutes, SD 4.3; *P*<.001). In contrast, the total evaluation time in the telemedicine group was longer than that in the conventional group (12.2 minutes, SD 3.5, vs 8.9 minutes, SD 4.3; *P*<.001). To evaluate the quality of interviews, the two groups were compared in terms of the 72-hour ED revisit rate. The analysis did not show a statistically significant difference in this rate between the groups (2/93, 2.7%, in the telemedicine group vs 0/105, 0.0%, in the conventional group; *P*=.50) (Table 1). The crude and adjusted mean difference estimates of the total exposure time and total evaluation time between the two groups are shown in Table 2. After adjusting for age, gender, triage level, educational status, and primary care physician level, we found that the total exposure time in the telemedicine group was 4.6 minutes shorter (95% CI −5.7 to −3.5, *P*<.001) than that in the conventional group. However, the total evaluation time in the telemedicine group was 2.8 minutes longer (95% CI −1.6 to −4.0; *P*<.001) than in the conventional group.

**Table 2:** Estimates of the crude and adjusted mean differences in the total evaluation time and total exposure time in minutes between the telemedicine and conventional groups.

Outcome	Telemedicine group (n=93), mean (SD)	Conventional group (n=105), mean (SD)	Mean difference estimate^a^			95% CI		*P* value	
			Crude	Adjusted^b^	Crude		Adjusted	Crude	Adjusted
Total exposure time	4.7 (2.4)	8.9 (4.3)	–4.2	–4.6	–5.2 to –3.2		–5.7 to –3.5	<.001	<.001
Total evaluation time	12.2 (3.5)	8.9 (4.3)	3.3	2.8	2.2 to 4.4		1.6 to 4.0	<.001	<.001

^a^The conventional group estimate was subtracted from the telemedicine group estimate.

^b^The model was adjusted for age, gender, triage level, educational status, and primary care physician level.

### Comparison of the Mean Scores of the Questionnaire Survey Between the Telemedicine Group and the Conventional Group

Table 3 compares the mean scores of the questionnaire survey between the telemedicine group and the conventional group. Regarding the quality of interpreting imaging and laboratory studies (Question 4), the mean scores for the telemedicine group and the conventional group were 4.7/5 and 4.5/5, respectively (*P*=.07). The scores in relation to acceptance, safety, and satisfaction with the telemedicine protocol were all 4.7/5. The overall patient satisfaction with ED visits did not significantly differ between the two groups (mean score 4.6 vs 4.5, *P*=.33). There were no intergroup differences in the other variables (Table 3).

**Table 3:** Numbers of respondents by point of the 5-point Likert scale and mean scores of the telemedicine and conventional groups.

Survey question	Telemedicine group (n=82)^a^						Conventional group (n=96)^b^							*P* value^c^	
	Response^d^, n (%)					Mean score		Response, n (%)					Mean score		
	1	2	3	4	5			1	2	3	4	5			
1. I am satisfied with the visit.	1 (1)	0 (0)	3 (4)	20 (24)	58 (71)	4.6		0 (0)	0 (0)	3 (3)	29 (30)	60 (63)	4.5		.33
2. I had enough time to tell the doctor about what happened to me.	1 (1)	0 (0)	1 (1)	21 (26)	59 (72)	4.7		0 (0)	0 (0)	4 (4)	29 (30)	63 (66)	4.6		.35
3. The doctor understood my presentation well.	0 (0)	0 (0)	6 (7)	20 (24)	56 (68)	4.6		0 (0)	0 (0)	6 (6)	29 (30)	61 (64)	4.6		.58
4. The doctor clearly explained evaluation and X-ray results to me.	0 (0)	1 (1)	3 (4)	16 (20)	62 (76)	4.7		0 (0)	2 (2)	8 (8)	25 (26)	61 (64)	4.5		.07
5. I could hear the doctor’s voice clearly.	0 (0)	0 (0)	2 (2)	23 (28)	57 (70)	4.7		0 (0)	1 (1)	5 (5)	24 (25)	66 (69)	4.6		.78
6. I had enough time to ask questions.	1 (1)	0 (0)	3 (4)	21 (26)	57 (70)	4.6		0 (0)	2 (2)	4 (4)	28 (29)	62 (65)	4.6		.48
7. I felt relaxed when I talked to the doctor.	1 (1)	0 (0)	3 (4)	19 (23)	59 (72)	4.6		0 (0)	1 (1)	7 (7)	21 (22)	67 (70)	4.6		.67
8. During the visit, I was not scared or stressed.	1 (1)	0 (0)	3 (4)	18 (22)	60 (73)	4.7		0 (0)	3 (3)	4 (4)	26 (27)	63 (66)	4.6		.27
9. I did not feel discriminated during the visit.	1 (1)	0 (0)	2 92)	17 (21)	62 (76)	4.7		0 (0)	0 (0)	4 (4)	25 (26)	67 (70)	4.7		.40
10. My privacy was well-protected.	0 (0)	1 (1)	6 (7)	19 (23)	56 (68)	4.6		0 (0)	0 (0)	7 (7)	27 (28)	62 (65)	4.6		.69
11. I think video interviews are acceptable.	0 (0)	0 (0)	2 (2)	22 (27)	58 (71)	4.7		N/A^e^	N/A	N/A	N/A	N/A	N/A		N/A
12. I felt safe during the video interview.	1 (1)	0 (0)	4 (5)	15 (18)	62 (76)	4.7		N/A	N/A	N/A	N/A	N/A	N/A		N/A
13. I am satisfied with the video interview.	1 (1)	0 (0)	2 (2)	18 (22)	61 (74)	4.7		N/A	N/A	N/A	N/A	N/A	N/A		N/A

^a^A total of 11 non-respondents in the telemedicine group were excluded.

^b^A total of 9 non-respondents in the conventional group were excluded.

^c^Analyzed using the Mann-Whitney U test.

^d^5-point Likert scale: 1. Strongly disagree. 2. Disagree. 3. Neutral. 4. Agree. 5. Strongly agree.

^e^Not applicable.

## Discussion

### Principal Results

This study shows that the double triage and telemedicine protocol in the ED could reduce physicians’ time of exposure to patients who pose a risk of COVID-19 transmission without compromising patient satisfaction. The time of direct exposure to individual patients in the telemedicine group could be 39% to 64% of that in the conventional group. During the COVID-19 pandemic, our protocol could effectively protect our health care workers from contracting infections in the ED. Moreover, wearing PPE has been shown to constrict mobility and vision and cause heat stress and dehydration, especially in hot weather [33]. This protocol could therefore alleviate the workload and stress of health care workers. However, the total evaluation time in the telemedicine group was longer than that in the conventional group; this may be due to the fact that physicians spent more time communicating with and providing explanations to patients. Our protocol provided a safer and more comfortable interview environment than the conventional method.

### Limitations

Our study has some limitations that must be addressed. First, it was a retrospective study using prospectively collected data. Confounding factors during this period, including policy changes, patient characteristics, medical resources, and laboratory examination, may have affected the study results. However, the patients in both the telemedicine group and the conventional group were relatively comparable. The policy in Taiwan and the criteria of reporting remained roughly unchanged, leading to small confounding effects of time and other policy factors. Second, our patients were relatively young, and telecommunication use was quite common among them; this explains their high familiarity with and acceptance of video interviews. Further studies should therefore investigate the feasibility and benefits of telemedicine among older adults. However, in our COVID-19 emergency clinic, a nurse was assigned to assist patients in receiving video calls if needed, and the patients’ families were allowed to accompany them in the clinic and could also help them use the TIS. These mechanisms facilitated the efficient functioning of the system. Third, in our study, 203 patients were excluded due to high TTAS levels. The feasibility of telemedicine for these patients was not investigated. Because these patients are usually in critical condition, face-to-face emergent management may be necessary in clinical scenarios. The possible application of telemedicine with critical patients should be further developed and examined. Fourth, the study was conducted at a single ED in Taiwan. Therefore, the generalizability of the novel model has not been confirmed. Furthermore, the cultural differences between Taiwan and other countries should be considered to ascertain the effectiveness of the proposed model.

### Comparison With Prior Work

The double triage method in our protocol facilitated the use of a TIS. The epidemic of an emerging infectious disease may cause a surge of patients in EDs due to clinical symptoms or fear of the disease. This may overwhelm the health care capacity of overcrowded EDs and aggravate the risk of cross infection in hospitals [34]. The CDC has suggested mandatory rapid, safe triage and isolation of patients with symptoms of COVID-19 in EDs for infection control. On admission, all patients should be surveyed about the presence of fever, symptoms of COVID-19, or contact with suspected COVID-19 cases. Patients with symptoms of COVID-19 should be isolated for examination in a separate, well-ventilated space [7]. In Taiwan, we adapted our response to the COVID-19 outbreak based on our experience with SARS in 2003, whereby early identification of patients suspected of having COVID-19 through “triage screening” could help prevent in-hospital transmission [34]. Our ED developed a double triage method to survey suspected patients with COVID-19 and separated them from others. Moreover, this double triage method helped identify patients eligible for telemedicine interviews. For suspected patients with COVID-19 in critical condition, physicians should immediately proceed to face-to-face evaluation and provide them with resuscitation. However, telemedicine interviews could be a safe alternative to face-to-face interviews for patients in stable condition. The protocol described in our study may provide a practical and feasible strategy for other EDs to improve their infection control measures.

Telemedicine has long been used to provide medical care in remote areas and has proved to be beneficial in infection control and management. The published literature demonstrates that telemedicine can increase access to care, with high patient satisfaction, improved outcomes, and reduced costs [35]. Furthermore, the Infectious Diseases Society of America has issued a position statement on telehealth that outlines the various uses of telemedicine and telehealth and supports the appropriate use of telehealth in clinical care, research, and education [36]. Some applications for infection control have been reported in the past decades [19,23], such as telemonitoring of asymptomatic individuals identified as case contacts during the Ebola virus disease outbreak in Africa in 2014. Other applications include caring for symptomatic cases that require isolation (Taiwan during the SARS epidemic in 2003 and the H1N1 influenza pandemic in 2009), tele-expert consultation, and tending patients without access to health care facilities (the MERS epidemic in Korea in 2015). In the current COVID-19 pandemic, many countries are accelerating their transformation to the virtual care interface [37]. Telemedicine, particularly video consultations, has been promoted and scaled up to reduce the risk of transmission. However, almost all existing evidence pertains to highly selected samples of outpatients with chronic, stable conditions. It is largely irrelevant to the current escalating situation that involves patients with an acute and potentially serious illness. Our study showed that telemedicine is feasible and can benefit both infection control and the provision of quality care for suggested COVID-19 patients. Our protocol involves the novel synchronous application of telemedicine for infection control.

The use of video interviews in our TIS had certain advantages over telephonic or conventional methods. Through face-to-face communication, we can directly confirm the identity of patients in routine practice and examine their general appearance and respiratory status. Moreover, the telemedicine system has a share function that can help physicians to simultaneously send high-quality images, laboratory data, and health information to their patients. Notably, our study results showed that the telemedicine group had a more favorable impression of the quality of images and interpretation of laboratory studies than the conventional group, although no statistical significance was observed.

Certain concerns exist about the feasibility and acceptance of telemedicine, especially in the context of the pandemic [24,27]. In this study, we evaluated patients’ perception and acceptance of telemedicine, and the results indicated that both groups showed similar overall satisfaction with the quality of interviews. With regard to the perception of stress, discrimination, and privacy, there were no significant differences between the groups. In fact, real-time video interviews may reduce patients’ anxiety and physicians’ stress.

### Conclusions

The implementation of a double triage and telemedicine protocol during the COVID-19 pandemic has high potential to improve infection control. Our study preliminarily validated a promising model in the ED to minimize physicians’ direct contact with non-critical suspected COVID-19 patients during evaluation. During the pandemic, this model could help protect critical medical personnel in the health care system from unnecessary exposure and further prevent overwhelming of the health care system.

## Appendix

Multimedia Appendix 1 Photographs of the physician's site (A) and the patient’s site (B) in the COVID-19 emergency clinic.

## Abbreviations

CDC: US Centers for Disease Control and Prevention
COVID-19: coronavirus disease
ED: emergency department
MERS: Middle Eastern respiratory syndrome
NTUH: National Taiwan University Hospital
PPE: personal protective equipment
RT-PCR: reverse transcription–polymerase chain reaction
SARS: severe acute respiratory syndrome
SARS-CoV-2: severe acute respiratory syndrome coronavirus 2
TIS: telemedicine interview system
TOCC: travel, occupation, contact, and cluster
TTAS: Taiwan Triage and Acuity Scale
