# Supplementary figures and images for: A Double Triage and Telemedicine Protocol to Optimize Infection Control in an Emergency Department in Taiwan During the COVID-19 Pandemic: Retrospective Feasibility Study
Source: J Med Internet Res. 2020 Jun 23;22(6):e20586. doi: 10.2196/20586 (PMC7313383; doi:10.2196/20586)

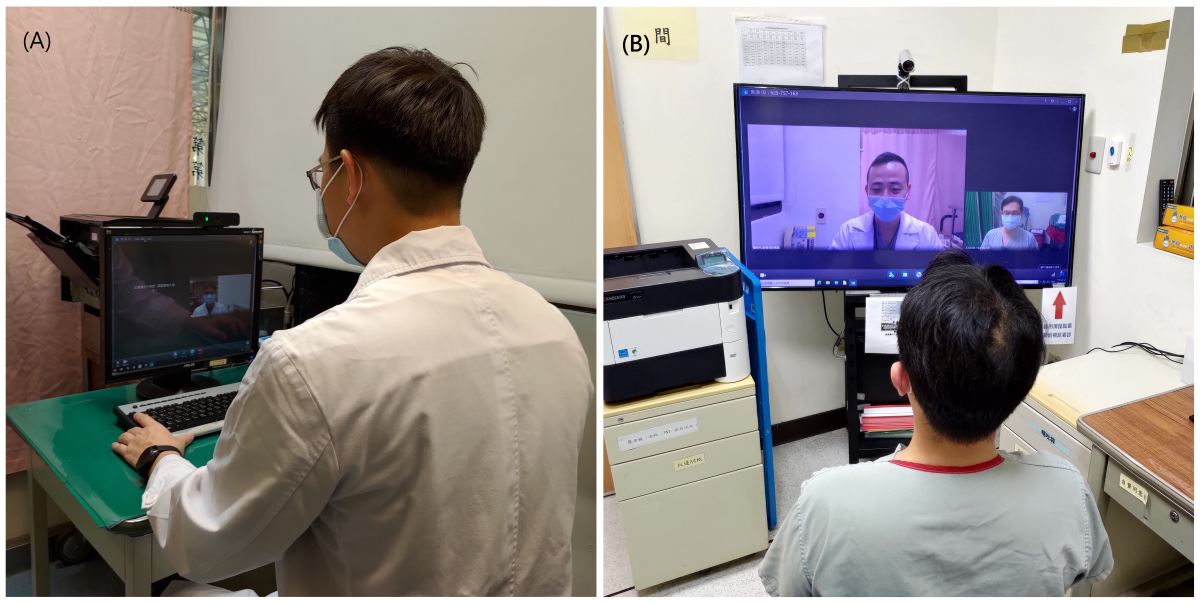

Supplement: Multimedia Appendix 1 [file jmir_v22i6e20586_app1.png]
